# Radionuclide Gas Transport through Nuclear Explosion-Generated Fracture Networks

**DOI:** 10.1038/srep18383

**Published:** 2015-12-17

**Authors:** Amy B. Jordan, Philip H. Stauffer, Earl E. Knight, Esteban Rougier, Dale N. Anderson

**Affiliations:** 1 Los Alamos National Laboratory, Los Alamos, NM 87545.

## Abstract

Underground nuclear weapon testing produces radionuclide gases which may seep to the surface. Barometric pumping of gas through explosion-fractured rock is investigated using a new sequentially-coupled hydrodynamic rock damage/gas transport model. Fracture networks are produced for two rock types (granite and tuff) and three depths of burial. The fracture networks are integrated into a flow and transport numerical model driven by surface pressure signals of differing amplitude and variability. There are major differences between predictions using a realistic fracture network and prior results that used a simplified geometry. Matrix porosity and maximum fracture aperture have the greatest impact on gas breakthrough time and window of opportunity for detection, with different effects between granite and tuff simulations highlighting the importance of accurately simulating the fracture network. In particular, maximum fracture aperture has an opposite effect on tuff and granite, due to different damage patterns and their effect on the barometric pumping process. From stochastic simulations using randomly generated hydrogeologic parameters, normalized detection curves are presented to show differences in optimal sampling time for granite and tuff simulations. Seasonal and location-based effects on breakthrough, which occur due to differences in barometric forcing, are stronger where the barometric signal is highly variable.

Predicting the travel time, window of opportunity for detection, and concentration of radionuclide gases from underground nuclear explosions (UNEs) is of considerable importance to the international community for monitoring for compliance with the Comprehensive Nuclear Test-Ban Treaty, which provides stipulations for sampling to help verify whether suspected explosions by signatory countries are nuclear in nature. This research investigates late-time seepage (weeks to months) of radionuclide gases that migrate under the influence of barometric pumping through explosion-enhanced fracture networks.

One of several gases that may be monitored under the treaty is ^133^Xe, which has a half-life of 5.2 days and is produced in UNEs, as well as naturally^1^ and in anthropogenic processes^2^,^3^,^4^. The independent yield of ^133^Xe is approximately 2 × 10^13^ Bq/kt^5^, released during the explosion along with other gases in the explosion cavity. Other gases of interest for monitoring under the CTBT include different isotopes of xenon (^131^ ^m^Xe, ^133^ ^m^Xe, ^135^Xe) and ^37^Ar, but only ^133^Xe is analyzed in this paper. As pressures drop after the detonation and return to ambient levels, the cavity may no longer support the overlying rock and a “chimney” zone of rubblized rock may form. Gases will likely homogenize in this region of high permeability and porosity immediately after the explosion, driven by convection from the heat of the explosion^6^. Experience from prior U.S. testing suggests that many UNEs did not vent gases at the surface immediately^7^; drill-back studies show evidence of the limited extent of transport of volatile components in the early times following explosions^8^, providing initial conditions for simulations of late-time seepage.

The driving force for late-time gas seepage through the unsaturated zone is thought to be barometric pumping from atmospheric pressure cycles^9^, a mechanism by which atmospheric pressure variations can draw subsurface vapor-phase contamination upwards. Gases are advected by pressure gradients following Darcy’s Law, both between the fracture and matrix material and strongly within fractures. During barometric lows, gases generally migrate towards fractures and upwards; during highs, atmospheric air is pushed into the subsurface. Concurrently, diffusion along concentration gradients spreads the trace gas of interest from contaminated to fresh air. Single-fracture and plane-parallel fracture models have produced good matches to tracer gas data using this conceptual model for gas migration, and have been used to predict UNE gas breakthrough timing and isotopic fractionation^6^,^10^,^11^,^12^. Such models are simple to build using numerical or analytical solutions^13^,^14^,^15^,^16^,^17^, and are extremely useful for understanding characteristics of barometrically pumped systems, but they neglect the effects of heterogeneous, anisotropic fracturing. Gas transport via barometric pumping through complex 3-D fracture networks has also been examined^18^. However, transport through fractures produced by a physics-based hydrodynamic explosion model has not been simulated before.

We present a sequentially-coupled hydrodynamic rock damage and gas transport model (Fig. 1) and investigate the effects of the fracture network on radionuclide gas migration and breakthrough timing and window of opportunity for detection. Simulations are presented of 1 kt UNEs in jointed granite and tuff, at depths of burial of 125, 250, and 390 m. In addition to nuclear explosion monitoring and treaty verification, our coupled model could be applied to other geophysical systems that produce fractures with subsequent flow, such as hydrofracturing, wastewater injection, mine explosions, and damaged rock zones around excavations. The gas transport results are relevant to other applications, such as radon and methane migration, soil vapor extraction for cleanup of contaminated sites, and landfill gas migration.

## Results

### Rock Damage

**Figure 2:**
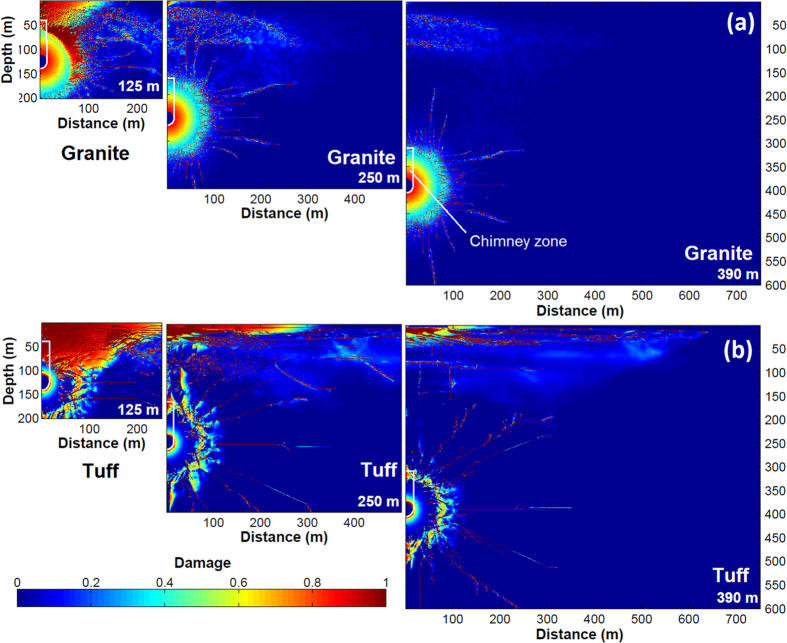
Damage results at 125, 250, and 390 m depth of burial. The chimney, which overlays the hydrocode-produced damage for transport calculations, is outlined in white. (**a**) Granite and (**b**) tuff.

Figure 2 shows results from the hydrocode simulations. The damaged area around the explosive charge is caused by material failure in compression. Moving outwards, the amount of compressive damage decreases until a point where tensile cracking begins, and total damage increases. Compressive damage around the charge for tuff is less than for granite, due to the higher saturation (discussed in the Methods section). As depth of burial decreases, a significantly damaged region near the surface is observed, generated by the interaction between the stress wave and the free surface.

### Gas Flow and Transport

To test the sensitivity of gas breakthrough to varying individual parameters, simulations were run with matrix permeability (*k*_*m*_), maximum fracture aperture (*δ*_*f,max*_), matrix porosity (*ϕ*_*m*_), and saturation uniformly spanning a range of possible values (25 runs for each varying parameter to span the range), with the other parameters fixed (Table 1). Additionally, to test the coupled effects of varying parameters, stochastic simulations were performed with all parameters allowed to vary simultaneously. 100 realizations were run for each scenario with parameters generated using Latin hypercube sampling over ranges given in Table 1. Matrix and chimney permeability (*k*_*m*_, *k*_*c*_) were sampled from a uniform distribution of log(*k*). Matrix and chimney porosity (*ϕ*_*m*_, *ϕ*_*c*_), *S*, and *δ*_*f,max*_ were sampled from uniform distributions. The location of the pressure signal used and the season of detonation for that location (either January or June) are given for each case in Table 1.

Because granite and tuff differ in hydrogeologic properties, different ranges were used for the stochastic simulations (MC1 and MC2 in Table 1); a comparison was also made for the fracture network alone by using the granite rock damage model with tuff-appropriate properties (case MC3), which is presented in the Supplementary Information online. To test the effect of the variability of the barometric pressure signal on gas transport (case MC4), pressure data from Anchorage and Honolulu were used, and simulations were started in January and June to test the effect of the season of detonation.

**Figure 3:**
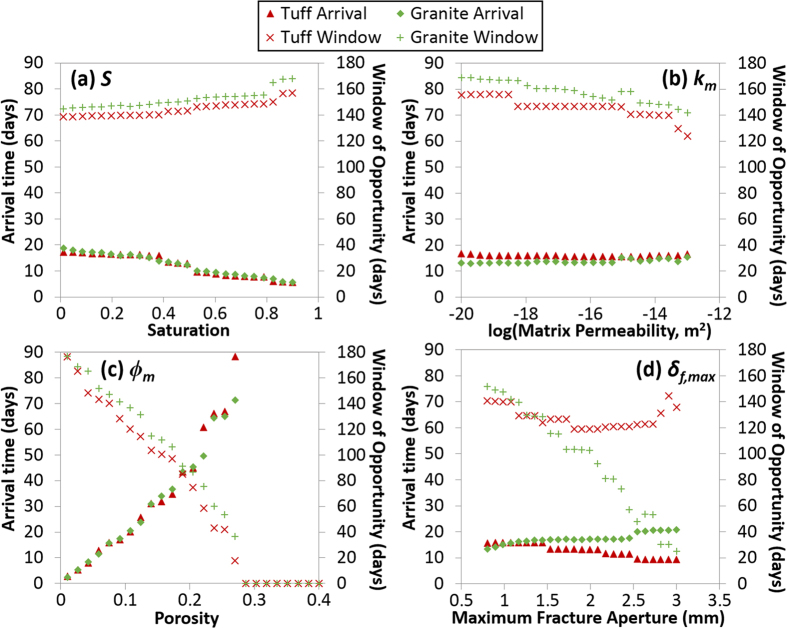
Gas transport sensitivity results with uniformly varying hydrogeologic properties for granite and tuff. Effect of varying (**a**) saturation, (**b**) matrix permeability, (**c**) porosity, and (**d**) maximum fracture aperture on ^133^Xe arrival time and detection window of opportunity.

The impact of parameters on gas arrival (days following detonation) and window of opportunity for detection are shown in Fig. 3. Gas arrival time represents the first concentration above a detection limit of 5 × 10^−22^ mol/kg air (0.6 mBq/m^3^)^19^. Although many realizations drop below the detection limit during high pressures, the window is computed based on first breakthrough until ^133^Xe decays permanently below the detection threshold. Full breakthrough curve plots for the sensitivity tests are shown in Supplementary Fig. S1 online.

Each panel of Fig. 3 shows the effect of a single parameter, varying uniformly across the x-axis range, with all other parameters held fixed. For both rock types, saturation and matrix permeability generate little difference in gas breakthrough, while porosity and maximum fracture aperture are significant. For example, for porosity (Fig. 3c), for both tuff and granite, arrival time gets larger (gas breakthrough is more delayed) as porosity increases from 0.01 to 0.4. Concurrently, the window of opportunity shrinks until gas arrival time is greater than about 90 days, at which point the gases do not arrive at all because concentrations have dropped below detectable limits due to radioactive decay. At this point, the window of opportunity becomes zero.

The results differ from previous studies that have used 2-D planar single-fracture models to analyze gas breakthrough. In particular, Sun and Carrigan^6^ and Jordan *et al.*^12^ found that increased fracture apertures cause faster arrival and longer detection windows. With the fracture network model, the results are more complicated (Fig. 3d): for granite, increased fracture aperture leads to slower arrival, lower concentrations, and significantly decreased window of opportunity for detection. For tuff, increasing maximum fracture aperture shortens arrival time, but the window of opportunity first narrows (despite the earlier arrival time) until a broad minimum is reached (1.75–2 mm) then increases.

**Figure 4:**
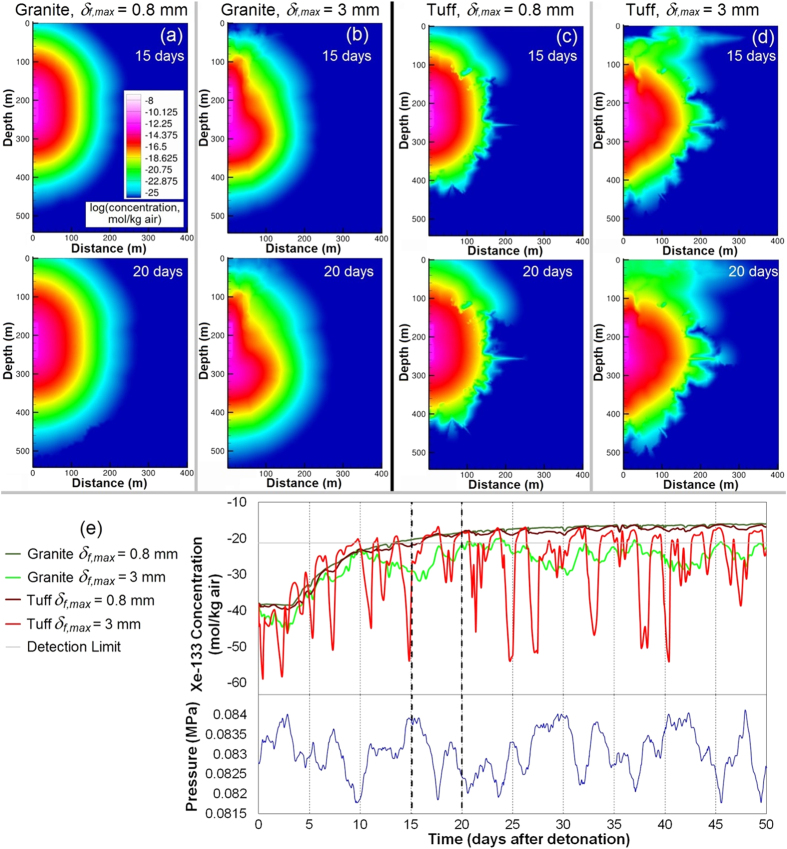
Comparison between granite and tuff at the minimum (0.8 mm) and maximum (3 mm) fracture aperture maximum (*δ*_*f,max*_). (**a–d**) Concentration plots for granite and tuff with varying *δ*_*f,max*_, shortly after a barometric high pressure period (15 days) and during a barometric low (20 days). (**e**) Breakthrough curves at the centerline surface node for granite and tuff with *δ*_*f,max*_ of 0.8 and 3 mm and barometric pressure for the first 50 days.

The counterintuitive results for increasing fracture aperture can be explained by two factors that apply for both rock types: increased lateral migration of ^133^Xe, which tends to decrease near-surface fluxes; and increased variability of surface concentration as a result of barometric fluctuations. Figure 4 shows ^133^Xe concentrations and breakthrough curves following low and high barometric pressure for granite and tuff for the smallest and largest fracture aperture cases in the sensitivity study. Increased lateral migration (Fig. 4a–d) and concentration variability (Fig. 4e) with increasing fracture aperture are evident. The key difference between rock types is greater damage near the surface for tuff (Fig. 2, 250 m), which leads to deeper access to contaminated air for a given barometric low pressure, as well as deeper propagation of the fresh air front during barometric highs. The effect of deeper breathing for tuff with increased *δ*_*f,max*_ overwhelms the lateral spreading effect, leading to the previously found positive correlation between fracture aperture and ^133^Xe breakthrough time. The differences in tensile damage (cracking) shown in Fig. 2 also affect the gas transport results, where granite shows a more homogeneous, diffusive appearance while for tuff, gas migration preferentially follows the strongly damaged radial fractures. These results underscore the importance of the layout of the fracture network in modeling gas migration.

**Figure 5:**
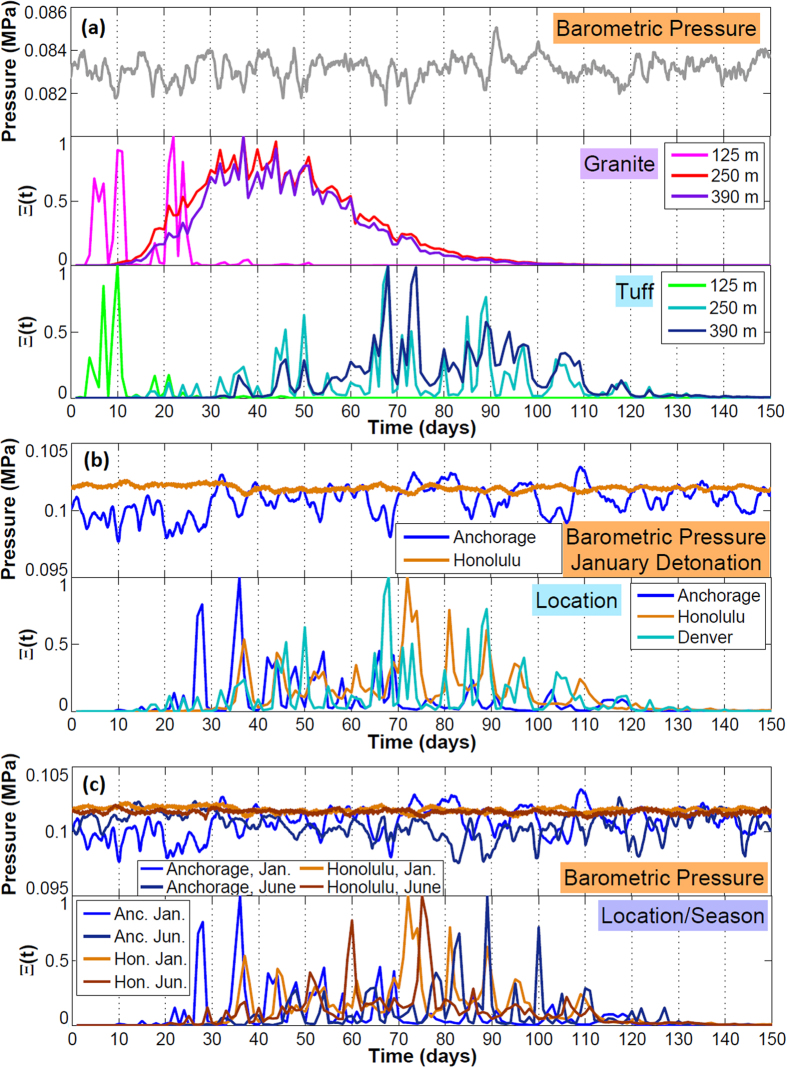
Normalized detection probability plots from stochastic simulations. (**a**) Barometric pressure for Denver, January; normalized detection probability, Ξ (*t*), for granite and tuff at all depths. (**b**) Barometric pressure for Anchorage and Honolulu, January; Ξ (*t*) for tuff (250 m) at all locations. (**c**) Barometric pressure for January and June detonations; Ξ (*t*) for tuff (250 m) at two locations and seasons.

Figure 5 summarizes the results of the stochastic simulations with daily-normalized detection probability curves, Ξ (*t*). These are one metric for optimizing sampling for gases based on the highest concentrations at the ground surface, with every realization treated as equally probable. The plots in Fig. 5 are to be interpreted as presenting information about the relative timing of concentration maxima for all realizations, independent of detection limit or absolute concentration. As such, these are not breakthrough curves. To produce the Ξ (*t*) detection probability curves, the concentrations at all output nodes along the top surface within each day are summed, weighted by the associated cell surface area and timestep length (Δ*t*_*s*_) in hours:


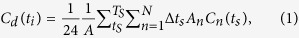


Where *C*_*d*_ is the daily sum of concentration on day *t*_*i*_; *t*_*s*_ is the timestep and *T*_*s*_ is the number of timesteps in the output history file within one day; *n* is the node along the top boundary, and *N* is the number of output nodes; *A*_*n*_ is the surface area of the cell; *A* is the total surface area of the domain; and *C*_*n*_(*t*_*s*_) is the concentration of that node at that timestep. This daily sum is then scaled for the whole time series by the maximum summed *C*_*d*_ for the realization *r*:


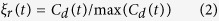


where each ξ_*r*_(*t*) is the scaled value of the daily concentration sum for day *t*, realization *r*. The time series ξ_*r*_(*t*) for each realization are summed to produce the daily total across all realizations:


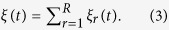


ξ is re-scaled to a maximum of 1 to produce the normalized decision curve:


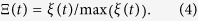


The scaling within each realization ensures that Ξ (*t*) is not dominated by high-mass simulations. Breakthrough curves for all stochastic simulations of ^133^Xe are given in the Supplementary Information (Figs S2–4).

Shallow explosions break through the fastest. The normalized curves are dominated by the oscillating barometric pressure (falling barometric pressure produces concentration increases, and vice versa); for a future event with unknown barometric pressure signal, the curve should be interpreted as providing general peak timing information, while actual sampling should occur based on a falling barometer^20^.

For granite, there is little difference between the 250 and 390 m best-guess sampling window, although earlier barometric pressure lows (20–30 days) have higher concentrations for the shallower case than the deeper case. For tuff, the 250 and 390 m cases are more spread out and dominated by barometric pressure. For both granite and tuff, the 125 m case has high probability peaks within 5–12 days. For granite, 100% of realizations produce gas breakthrough above the detection limit; for tuff, there is 100% breakthrough at 125 m, 98% at 250 m, and 21% at 390 m.

For a January detonation, cases with greater pressure variance have an earlier normalized concentration maximum. The Honolulu case has a maximum likelihood of detectability after Denver’s (72 versus 68 days, respectively), while the maximum for Anchorage in January falls at ~37 days. For Anchorage in June, after the more barometrically quiescent summer, the maximum falls at ~89 days. For Honolulu, 69–72% of simulations have breakthrough (depending on season), while for Anchorage it is 98–99%. For Anchorage, the season of detonation makes a considerable difference in the timing of maximum normalized breakthrough (Fig. 5c). For Honolulu, where the barometric pressure signal is more consistent throughout the year, the season of detonation makes little difference in ^133^Xe breakthrough, although the timing of the maximum of the normalized detection curve, Ξ(*t*), is dependent on the specific timing of storms throughout the year.

**Figure 6:**
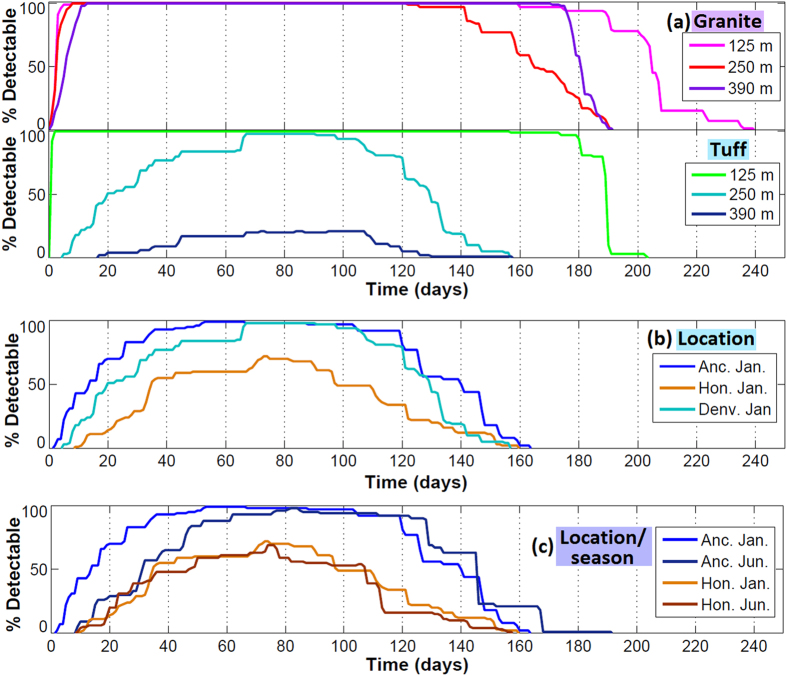
Percent of stochastic simulations with detectable ^133^Xe by day after the detonation. (**a**) Different rock types (granite and tuff) and depths of burial, for a January detonation with Denver pressure data. (**b**) Different locations, for a January detonation, in tuff at 250 m depth of burial. (**c**) Different locations and seasons, January and June detonations in Anchorage and Honolulu, in tuff at 250 m depth of burial.

For the cases shown in Figs 5 and 6 shows the percent of simulations with detectable ^133^Xe (based on a detection limit of 5 × 10^−22^ mol/kg air or 0.6 mBq/m^3^) per day. For these plots, ^133^Xe is considered potentially detectable between first breakthrough date and final drop below the detection limit, while actual concentrations may drop below the detection limit after first breakthrough during barometric high pressures (Supplementary Fig. S2–4 online). While the normalized detection probability curves in Fig. 5 represent the optimal time to sample to capture the maximum concentration of gases (with each realization treated as equally probable), these curves indicate more broadly when the stochastic simulations predict the greatest likelihood of detectable concentrations. The shallow tuff and all granite cases have a large time range where all simulations indicate potential detectability based on surface soil concentration. As before, actual sampling times would be optimized during barometric low pressure events.

Model assumptions (e.g., axisymmetric, isothermal) and uncertainties in key conditions (e.g., initial radionuclide gas distribution, chimney height) require further study, but these comparative analyses illustrate key physical processes that impact the travel time of gas seepage from UNEs under barometric pumping with gas diffusion. These models allow sensitivity studies to highly uncertain parameters, discussion of the effects of some of the major mechanisms that drive late-time seepage (i.e., barometric pumping), and evaluation of primary issues in the conceptual model such as whether the fracture network needs to be accurately simulated. Because of the great uncertainties in parameters, conceptual models, and processes, the results cannot be used at this time for accurate predictions of the timing, although they can be used for comparative predictions of timing. The results strongly demonstrate the importance of including the fracture network produced by the explosion in models of gas migration; the impact of the barometric forcing signal on the relative timing of gas arrival; and the effects and sensitivities to varying hydrogeologic parameters. Of these three issues, only the latter has been studied significantly by others^6^,^10^,^21^.

## Methods

The extent of rock damage for each type of material and depth of burial is simulated in a 2-D axisymmetric model using the CASH (CAmpbell-SHashkov) hydrocode^22^ with computational meshes containing ~3.3 million nodes. CASH was selected as the working code to conduct the modeling because it contains all the necessary capabilities for underground explosion fracture analysis. A visco-plastic continuum fracture material model was used^23^, which contains a rate-sensitive directional continuum fracture solver, combined with a dilatational friction solver; it introduces a dynamic overstress in high strain rate regimes. The shear and tensile strength material properties are stochastically seeded to represent pre-existing heterogeneities.

**Figure 7:**
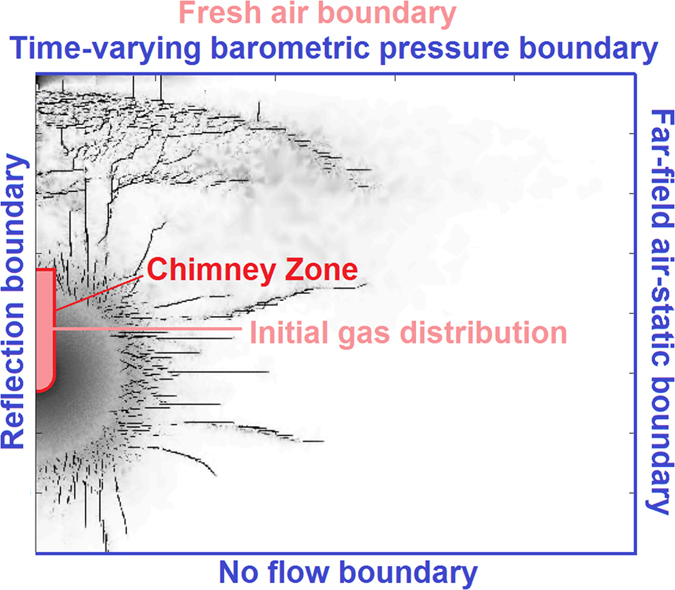
Simulation boundaries and initial gas distribution.

The fracture patterns calculated by the hydrocode are those generated by the propagation of the initial shock wave though the rock material. The collapse of the chimney (which usually occurs at a much later time) would affect the fracture patterns located in the neighborhood of the chimney and close to the free surface directly above the explosion. In this analysis it was assumed that these fracture patterns remain unchanged, and the effect of the chimney collapse was taken into account by increasing permeability and porosity in a rubblized chimney zone (Fig. 7). With respect to the persistence and stability of mid- to far- field fracturing, there will be a settling period where the apertures of some of the fractures may diminish and some may even close down. These are phenomena occurring over longer periods of time that could be incorporated/quantified when validating the mapping between damage and permeability, which is the subject of future work.

The tuff model contains a generic Mie-Gruneisen equation of state to describe the volumetric behavior of the material. Because the model was validated against a test explosion that took place in a saturated layer, the rock damage model for tuff assumes a completely saturated condition, although the gas flow and transport model allows saturation to vary. For unsaturated granite, an equation of state was developed to simulate pore crushing phenomena in geologic material.

The hydrocode has been validated for each of the rock types using field seismic velocity data. For wet tuff, the validation comes from the Non-Proliferation Experiment, a 1 kt chemical explosion at Rainier Mesa (Nevada National Security Site) in 1993. For granite the validation is performed by comparison with waveforms from the Source Physics Experiment. The model has not been rigorously tested for sensitivity to uncertain parameters (e.g., rock density, Young’s modulus, etc.).

The Finite Element Heat and Mass transfer code (FEHM) is used to simulate gas migration through the explosion-generated fracture networks in the unsaturated zone. FEHM is freely available (https://fehm.lanl.gov) and has been used for many applications, including nuclear waste disposal, contaminant transport, geothermal energy, and more^24^,^25^,^26^. Solutions to the governing equations of energy, mass, and momentum conservation are approximated in FEHM using the control volume finite element method with a Newton-Raphson iterative solver. It is assumed that Darcy’s Law is valid for all phases. Transport of solutes is governed by the advection-dispersion equation^27^. FEHM is regularly benchmarked against analytical solutions for flow and transport processes following all code modifications.

The 2-D radial simulation layout and boundary conditions for the FEHM flow and transport model are shown in Fig. 7. Radial symmetry allows 3-D volumes to be simulated while reducing computational burden. 2-D radial symmetry further means that fracture patterns are radially symmetric, a decent approximation of 3-D detonation fracturing. The boundaries are a no-flow bottom (e.g., a water table or low-permeability unit), fixed air-static pressure at the outer lateral boundary, and barometric pressure at the surface. The upper surface is a fresh air boundary for the transport calculations and a transient barometric pressure boundary with 2 hour timesteps (this limits the maximum timestep in the flow calculation to 2 hours, although generally the tracer timesteps are shorter, with initial tracer timestep of 0.24 hr upon each flow timestep). Water is present but immobile in the matrix, with saturation fixed to the values presented in Table 1 depending on the scenario.

The barometric data were selected from Denver, CO; Anchorage, AK; and Honolulu, HI (2013; available at http://www.ncdc.noaa.gov/data-access/land-based-station-data/land-based-datasets/quality-controlled-local-climatological-data-qclcd). These locations were selected because their barometric data represent a range of variability, from quiescent (Honolulu) to large-amplitude variability (Anchorage). The simulations are run with barometric pumping for 30 days prior to the detonation to initialize subsurface pressure. The simulations are isothermal as only late-time gas seepage is considered. Away from the cavity and chimney zones, gas equilibrates quickly to the rock temperature due to low total heat capacity^28^. This would underestimate the transport of radionuclides due to early-time, near-field convection and heat-pipe effects^6^, but our focus is on the effect of fracture networks on transport by barometric pumping alone. The radionuclide gas is initially homogeneously mixed in the chimney zone, a reasonable assumption considering convection and gas displacement from chimney collapse at early times^29^. The quantity of ^133^Xe initially emplaced in the chimney zone is based on the independent yield of a 1 kt UNE^30^. Because these simulations ignore production of ^133^Xe from parent radionuclides – which produces several orders of magnitude more ^133^Xe than the independent yield^5^—the actual concentrations are underreported here. The conclusions presented above regarding the effects of the fracture network on gas transport are unaffected, however.

The rock damage models end well before chimney collapse, while the transport models begin after ground motion has ceased. Therefore, we make assumptions about chimney height based on cavity radius. We use a chimney height of 84 m for all simulations, which is not expected to be identical for all rock types and depths of burial. The “rubblization” factor of the chimney is an uncertain parameter in our simulations, but it is assumed that the chimney is made up of large pieces of rock and that permeability and porosity are enhanced.

The fractured material and surrounding matrix are modeled as a dual permeability medium^31^. In a dual permeability model, every node is composed of two overlapping continuua, i.e., fracture and matrix material. Flow and transport are accounted for between matrix-fracture, fracture-fracture, and matrix-matrix material. The use of a dual permeability model for these simulations is key because it would be difficult to model fractures individually with enough resolution to cover the fracture-scale processes (sub-mm) across the spatial scale (250 to 750 m) of these simulations, and because dual-porosity models do not handle matrix-matrix transport^31^. In FEHM the user may specify particular nodes as dual continuum, while the rest of the domain behaves as single or equivalent continuum material.

The input parameters to the dual permeability model are the volume fraction of a cell that is composed of fracture material and the length scale between the centerline of the fracture and the center of the matrix material (*d*_*fm*_). Between the fracture and matrix material, the fluid transfer term (gas or liquid) is:


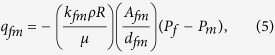


Where *k*_*fm*_ is the permeability between the two differing media (calculated as the harmonic mean of the fracture and matrix permeabilities); *ρ* and *μ* are fluid density and viscosity, respectively; *R* is a relative permeability term (set to 1 for the gas phase simulations performed here, where water is immobile); *A*_*fm*_ is the area separating the fracture and matrix; and *P*_*f*_ and *P*_*m*_ are pressures in the fracture and matrix, respectively. Between like material (matrix-matrix and fracture-fracture), flow and transport are computed normally^27^ but modified by a decreased connected area that reflects the fraction of cell volume taken up by the other material.

The numerical meshes for each scenario (rock type, depth of burial) are developed using the Los Alamos Grid Toolbox (http://lagrit.lanl.gov), with automatic mesh refinement where the rock is more highly damaged. Additional details are provided in the Supplementary Information (Fig. S5).

The matrix material is partially saturated with variable, immobile water content; fractures are assigned a fixed saturation of 0.01. Pore water acts as a source and sink of gas through Henry’s law partitioning:


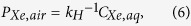


where *P*_*Xe,air*_ is the partial pressure of ^133^Xe in air (bar), *k*_*H*_ is the Henry’s law constant for ^133^Xe (0.0043 mol kg^−1^ bar^−1^)^32^, and *C*_*Xe,aq*_ is the aqueous concentration of ^133^Xe. Transport in the vapor phase is much more rapid by advection and diffusion than transport in the aqueous phase. The primary mechanism by which Henry’s law partitioning affects the bulk movement of ^133^Xe is when the mobile trace gas partitions into water in the fresh pores, and a subsequent barometric cycle cleans out the tracer in the pore, and ^133^Xe partitions back into the vapor from the aqueous phase to achieve equilibrium partial pressure.

The effective gas diffusivity is an important uncertain parameter that the gas migration results are sensitive to^6^. We use the Millington-Quirk approach^33^ to calculate the tortuosity (*τ*) for the porous matrix material as well as the fracture material, although the empirical relation is not validated for fractures:


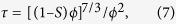


where *ϕ* is porosity and *S* is saturation. The effective diffusivity is *τD*_*0*_, where the free-air diffusivity (*D*_*0*_) for ^133^Xe is 1.24 × 10^−5^ m^2^/s^30^. The approximation for fractures is appropriate because within the fractures only, advection due to pressure gradients established by barometric cycling moves gases more effectively than diffusion. Furthermore, because the fractures are always set to saturation *S* = 0.01 and porosity of 1, there is no variation in effective diffusivity within the fractures between realizations (unlike the matrix material).

One of the key difficulties in integrating the rock damage and transport codes is linking the “damage” parameter to fracture and matrix properties. The gas transport results are very sensitive to this translation; however, for these sensitivity and stochastic analyses we use a simple linear mapping between damage and fracture aperture (Supplementary Fig. S6 online). Additional details are provided in the Supplementary Information. While further research into a realistic mapping is necessary to improve the accuracy of predictions of gas breakthrough, these comparative results provide important insights into the necessity and sensitivity to including a physics-based fracture network in gas migration models from UNEs.

## Additional Information

**How to cite this article**: Jordan, A. B. *et al.* Radionuclide Gas Transport through Nuclear Explosion-Generated Fracture Networks. *Sci. Rep.* **5**, 18383; doi: 10.1038/srep18383 (2015).

## Supplementary Material

Supplementary Information

## Figures and Tables

**Figure 1:**
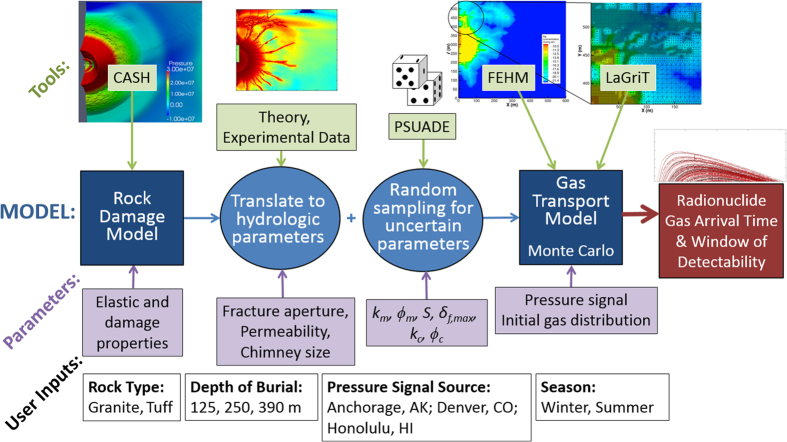
Schematic of the integrated model. The model sequentially couples a hydrodynamic rock damage code to a gas transport simulator via a translation between damage results and hydrogeologic parameters and stochastic sampling for uncertain hydrogeologic parameters.

**Table 1:** Ranges sampled for the sensitivity and stochastic simulations.

Case	Rock	Pressure Forcing	Depth (m)	Matrix *ϕ*_*m*_	*S*	Matrix *k*_*m*_ (m^2^)	*δ*_*f,max*_ (mm)	Chimney *ϕ*_*c*_	Chimney *k*_*c*_ (m^2^)
Sens.	Tuff and Granite	Denver, Jan.	250	0.01–0.4 0.36*	0.01–0.9 *0.07*	10^−20^–10^−13^ *1.5 *×* 10*^−*14*^	0.8–3*0.9*	*0.4*	*3.7 *×* 10*^−*12*^
MC1	Granite	Denver, Jan.	125, 250, 390	0.001–0.01	0.01–0.5	10^−19^–10^−14^	0.8–3	0.4–0.5	10^−12^–10^−10^
MC2	Tuff	Denver, Jan.	125, 250, 390	0.05–0.4	0.5–0.9	10^−18^–10^−13^	0.8–3	0.4–0.5	10^−12^–10^−10^
MC3	Granite	Denver, Jan. Anchorage,	250	0.05–0.4	0.5–0.9	10^−18^–10^−13^	0.8–3	0.4–0.5	10^−12^–10^−10^
MC4	Tuff	Honolulu;Jan., June	250	0.05–0.4	0.5–0.9	10^−18^–10^−13^	0.8–3	0.4–0.5	10^−12^–10^−10^

^*^Base case parameters for the sensitivity study ranges (Sens.) are given in italics.
