# Supplementary material for: Radionuclide Gas Transport through Nuclear Explosion-Generated Fracture Networks
Source: Sci Rep. 2015 Dec 17;5:18383. doi: 10.1038/srep18383 (PMC4682097; doi:10.1038/srep18383)
Supplement: Supplementary Information [file srep18383-s1.pdf]

Supplementary Information

Radionuclide Gas Transport through Nuclear Explosion-Generated Fracture Networks

Amy B. Jordan[1*], Philip H. Stauffer[2], Earl E. Knight[2], Esteban Rougier[2], and Dale N. Anderson[2]

[1] *Neptune and Company, 1505 15th St., Los Alamos, NM 87545*

[2]*Los Alamos National Laboratory, Los Alamos, NM 87545*

[*] *Corresponding author, Tel: 505-662-0707; Email: ajordan@neptuneinc.org*

This file contains:

Supplementary Figures S1-S6

Supplementary Text: Additional Gas Transport Numerical Model Description

Supplementary Figures S7-S8

Supplementary Text: Hydrocode Material Model Validation Data

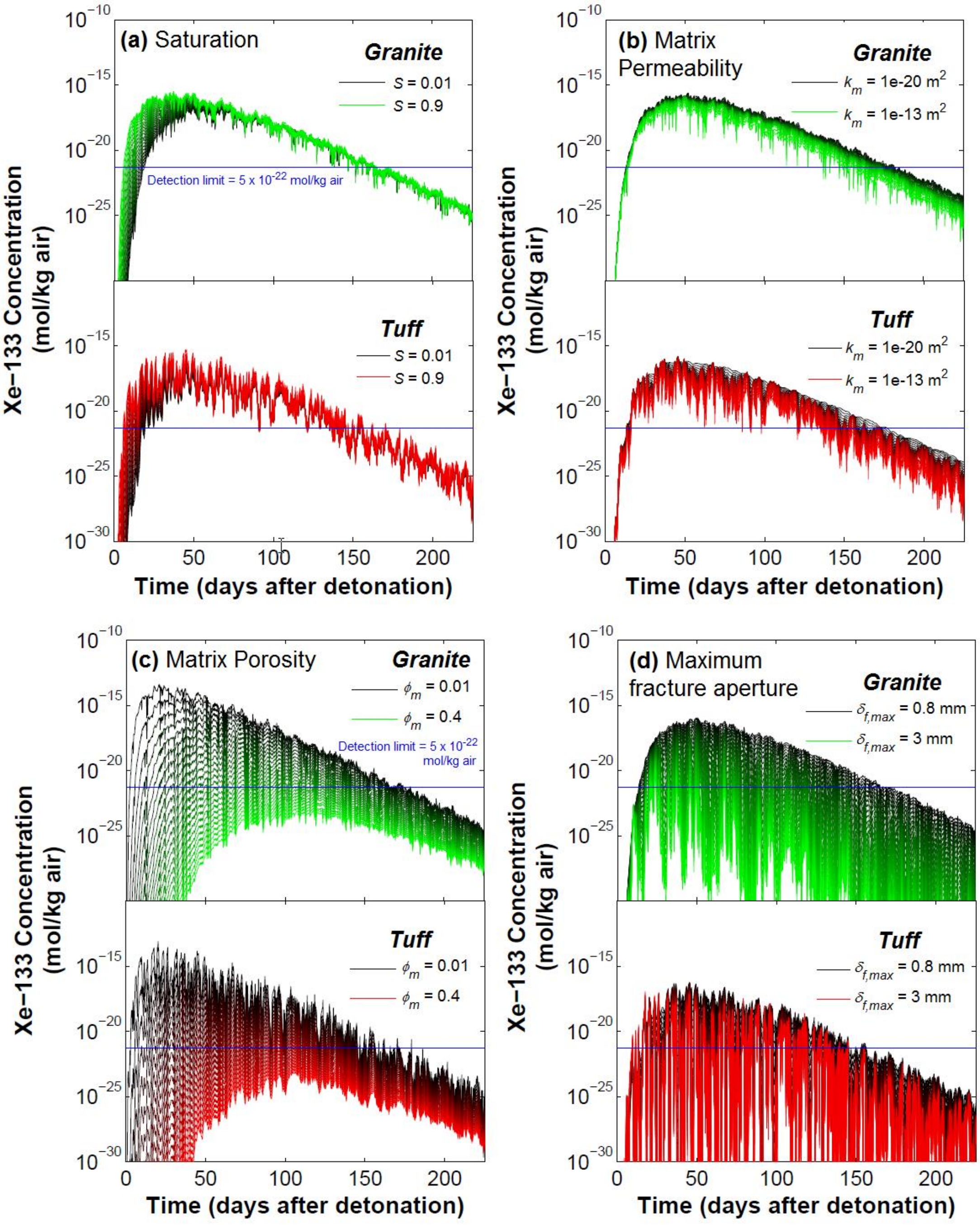

Figure S1. Sensitivity simulation breakthrough curves at the centerline surface node for granite and tuff, 250 m depth of burial. A detection limit of 5×10^{-22} mol/kg air (0.6 mBq/m^3) is shown in blue. The breakthrough curves shown span the parameter ranges given in Table 1 (main text) and each figure legend, linearly varying from black (smaller) to color (larger) values.

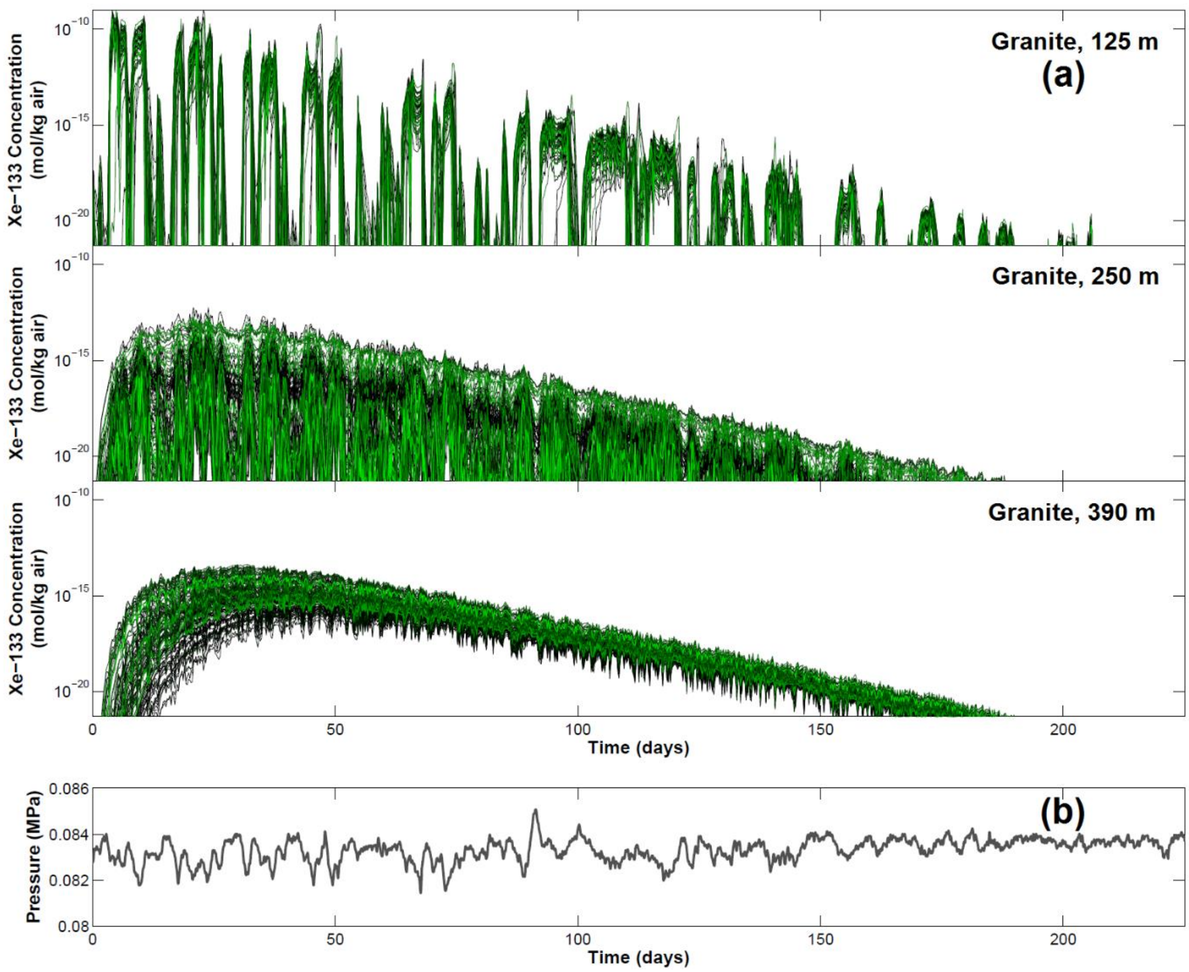

Figure S2. (a) Breakthrough curves at the centerline surface node for granite, for all realizations of case MC1 in Table 1 (main text), randomly colored by realization. The lower y-axis limit of each panel in (a) is an assumed detection limit of 5×10^{-22} mol/kg air (0.6 mBq/m^3). (b) Pressure data (Denver, January detonation).

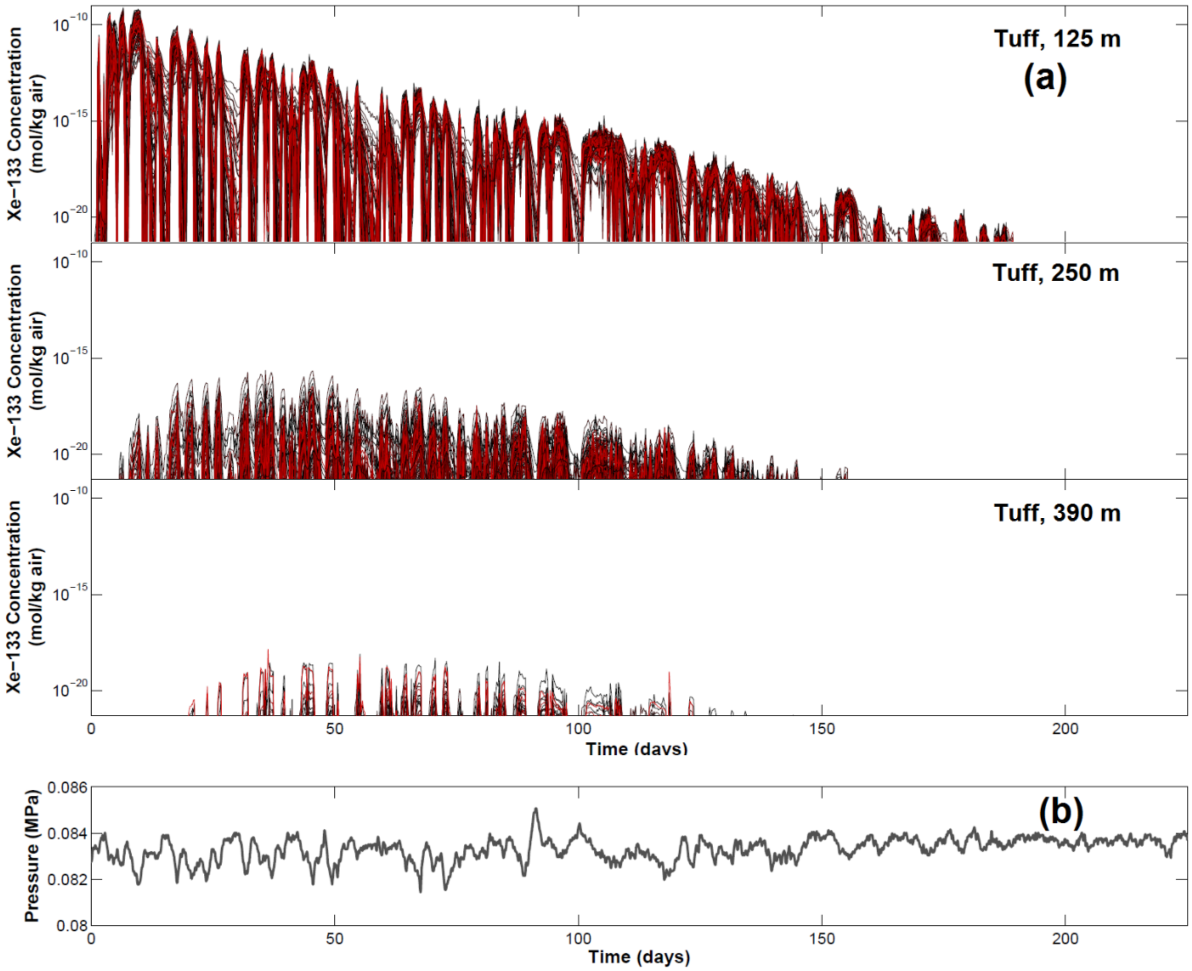

Figure S3. (a) Breakthrough curves at the centerline surface node for tuff, for all realizations of case MC2 in Table 1 (main text), randomly colored by realization. The lower y-axis limit of each panel in (a) is an assumed detection limit of 5×10^{-22} mol/kg air (0.6 mBq/m^3). (b) Pressure data (Denver, January detonation).

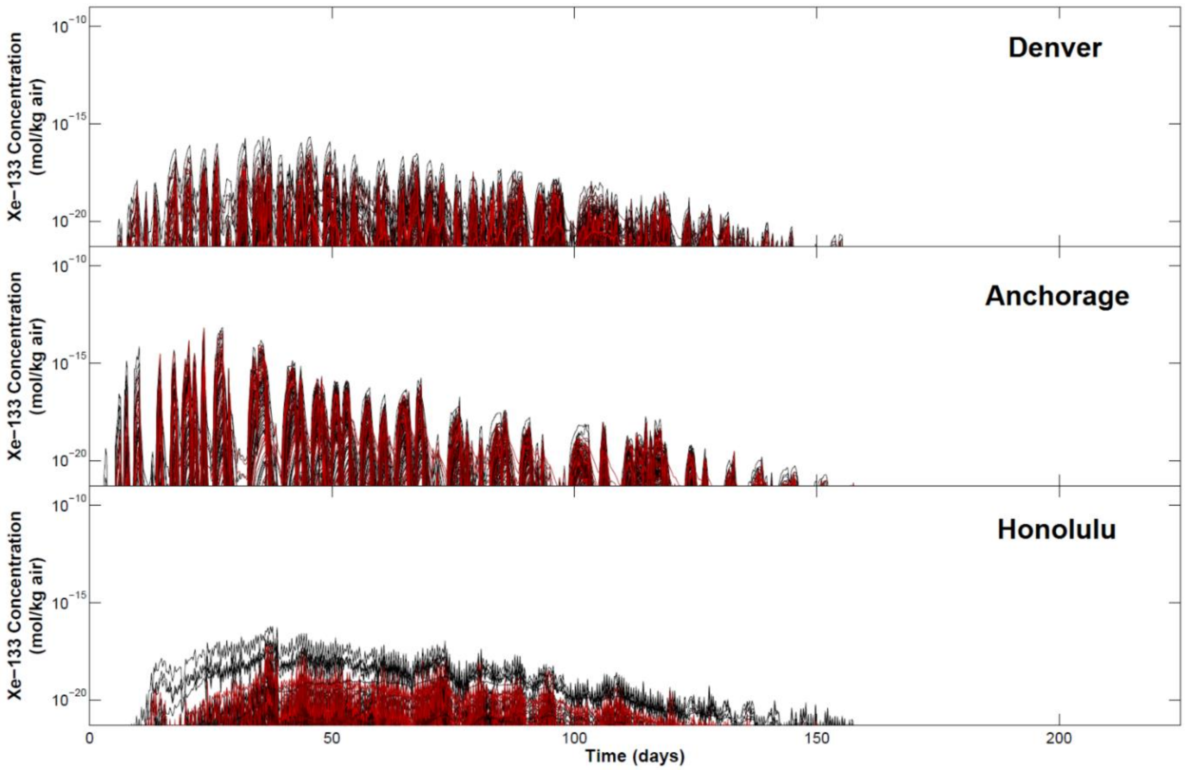

Figure S4. Breakthrough curves at the centerline surface node for tuff at 250 m depth of burial, for all realizations of case MC2 in Table 1 (main text), with varying barometric pressure signal based on data from three locations (Denver, Anchorage, and Honolulu), January detonation, randomly colored by realization. The lower y-axis limit of each panel in is an assumed detection limit of 5×10^{-22} mol/kg air (0.6 mBq/m^3).

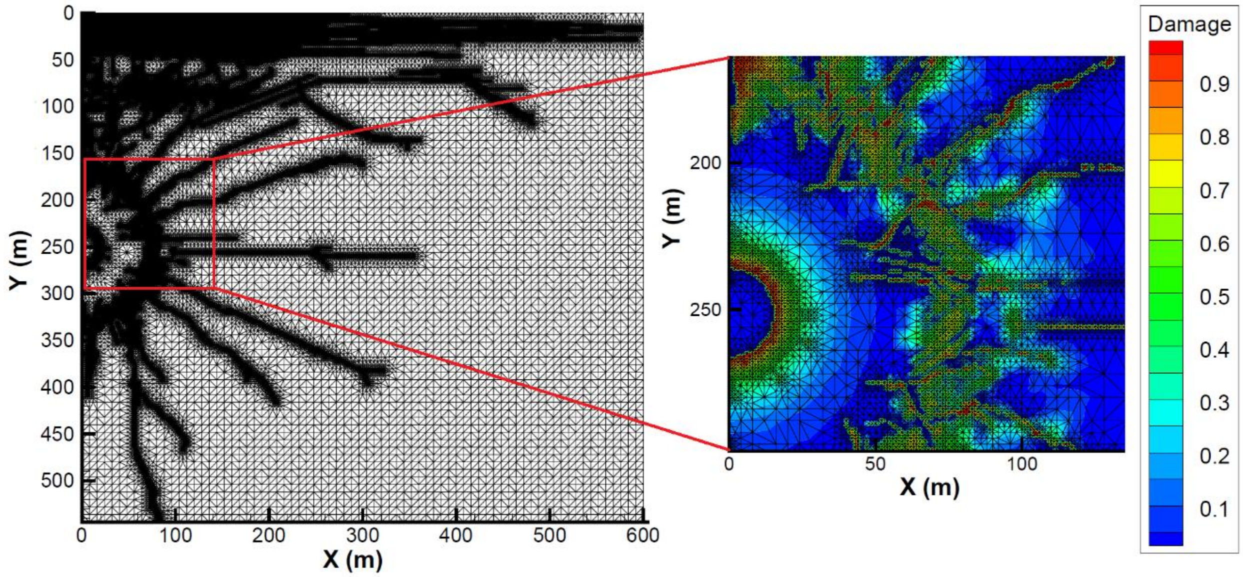

Figure S5. Mesh for the 250 m depth of burial tuff case, showing refinement (down to 1 m node spacing) for more highly damaged nodes.

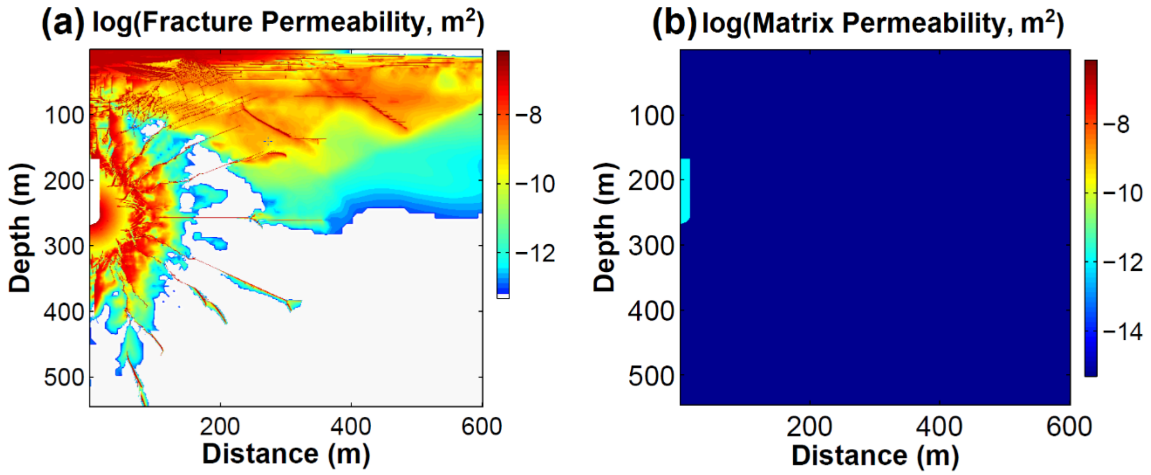

Figure S6. (a) Fracture permeabilites for GDKM nodes for one realization with $\delta_{f,max}$ = 1.41 mm. Note that these permeabilities represent only a tiny fraction of the node; the rest is composed matrix material. (b) Matrix permeability for all nodes. Outside the cavity/chimney zone, matrix permeability is 4.89 x 10^{-16} m^2 for this realization.

Additional Gas Transport Numerical Model Description:

The numerical gas flow and transport model used for this research was the Los Alamos National Laboratory (LANL) porous media flow and transport simulator FEHM. Figure S5 shows a sample grid (for tuff, 250 m depth of burial) showing mesh refinement. There are issues involved in the upscaling of damage from the hydrocode model meshes (~3.3 million nodes; minimum node spacing 10 cm) to the FEHM meshes with significantly fewer nodes (~40,000–65,000; minimum node spacing 1 m) for flow and transport calculations, as well as converting parameters to the dual permeability model. Averaging damage to upscale would not be appropriate for fracturing, where the dominant value in a region is the maximum and the average would result in greatly underpredicting the flow. The method employed in translating from the damage grid to the FEHM mesh is a nearest-neighbor type approach where Voronoi regions are generated around the FEHM mesh and the damage mesh, and the value assigned to the FEHM node is the damage for the damage Voronoi region with the greatest overlapping area.

The mapping used for this study from hydrocode damage results to gas flow and transport parameters is the following. We select the maximum fracture aperture ($\delta_{f,max}$), an uncertain parameter, and map it to the maximum damage; the lower cutoff for dual permeability (i.e., fractured) nodes is set to the fracture aperture (δ_f) that produces a fracture permeability (k_f) of 10^{-13} m^2 where $k_f = \delta_f^2/12$. Individual fracture permeability is assigned to the fracture nodes in the generalized dual permeability model within FEHM (GDKM) while the matrix component of the nodes have permeability k_m. Nodes with more damage than the cutoff are assigned to the dual permeability model, where the fracture component of the node has permeability of $k_f = \delta_f^2/12$ and matrix permeability of k_m. Nodes with less damage are considered “undamaged” and assigned k_m, where k_m represents an equivalent continuum model permeability (i.e., any sub-grid scale

fracture permeability is included in the matrix permeability). The dual permeability model cutoff is arbitrary, but represents a moderate permeability between typical maximum fracture permeabilities for the simulations (e.g., ~10^{-7} m^2 for the very largest fracture dual permeability nodes) to typical undamaged effective matrix permeability values (10^{-20}–10^{-17} m^2). (Matrix permeabilities in the stochastic simulations are allowed to range higher, to up to 10^{-13} m^2, to represent unmodeled, sub-mesh scale fracturing in an equivalent continuum approach.) The cutoff improves computational efficiency by not assigning all nodes of the domain to the dual continuum model.

Figure S6 shows permeabilities for one realization of the tuff 250 m depth of burial case. In this realization, maximum fracture aperture is 1.41 mm and matrix permeability is 4.89 x 10^{-16} m^2. In Fig. S6a, fracture permeabilities are shown for GDKM (fractured) nodes only. The cavity/chimney zone and all nodes where damage converted to permeability is less than 10^{-13} m^2 are treated as an equivalent continuum with an effective "matrix" permeability k_m. In the GDKM nodes shown in Fig. S6a, the fracture permeabilities represent only an exceedingly small fraction of the node volume. The remainder of the node is the matrix permeability shown in Fig. S6b.

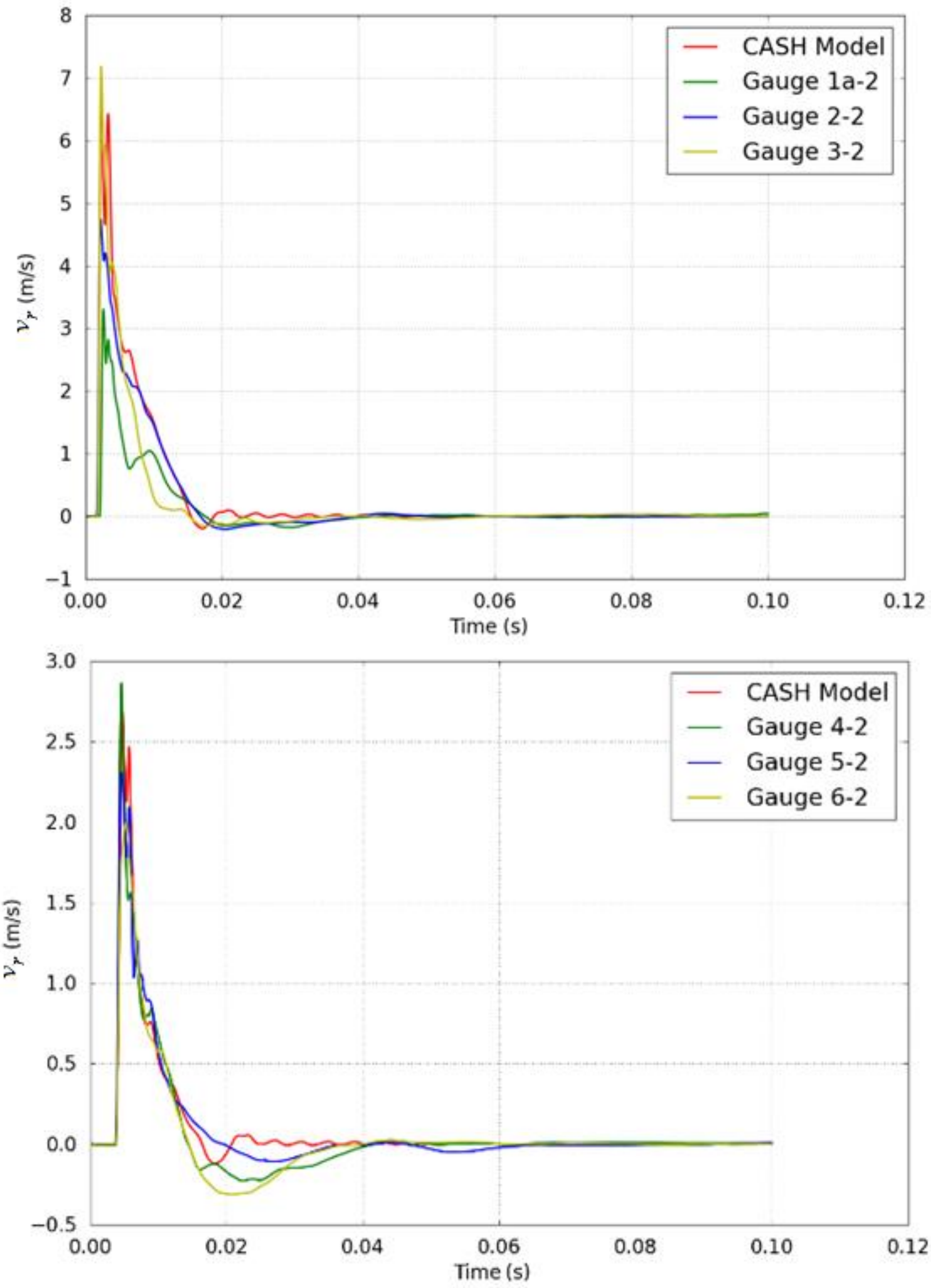

Figure S7. Comparison of the response obtained with CASH using the material model for granite versus free-field strong ground motion data provided by the experiment SPE-2.

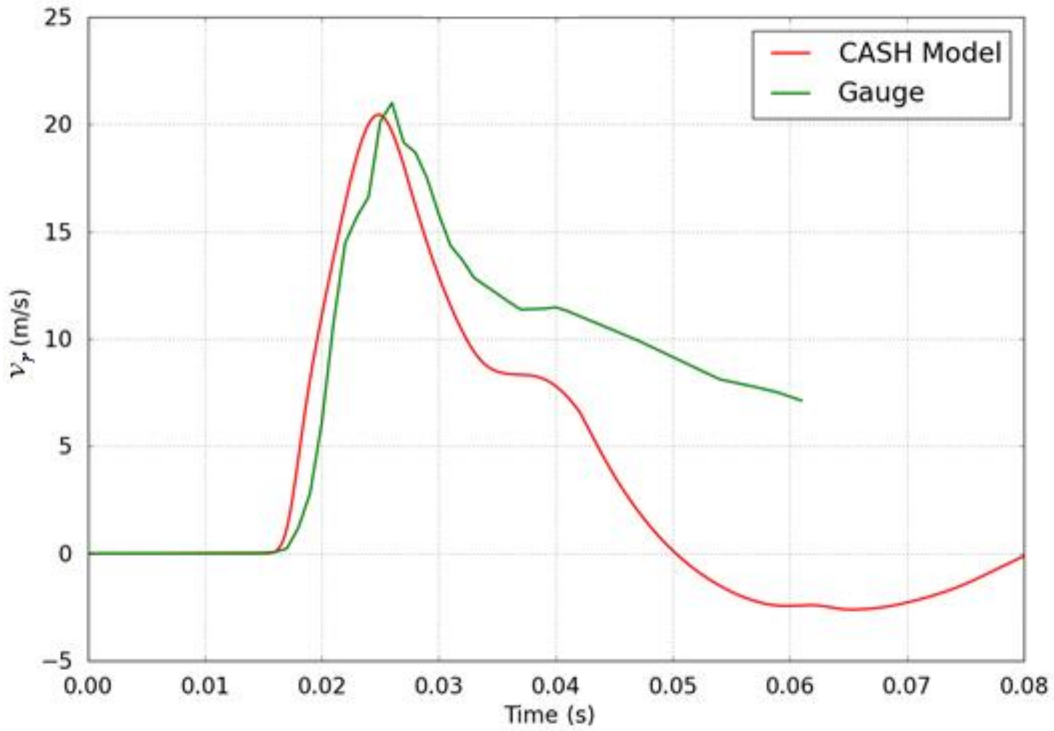

Figure S8. Comparison of the response obtained with CASH using the material model for tuff versus free-field strong ground motion data from the Non-Proliferation Experiment.

Hydrocode Material Model Validation Data:

The material model[7] utilized in this work was calibrated for both granite and saturated tuff by comparison of the CASH model results versus free field strong ground motion data from gauges placed around explosive experiments. For granite, the data provided by the Source Physics Experiment (SPE) was used as a reference. The comparison between the numerical results and the experiment for free field instruments located between a 10-m and 20-m range from the SPE-2 charge are shown in Figure S7.

For tuff, the experimental data was obtained from the Non-Proliferation Experiment (NPE). The comparison between the numerical results and a free-field gauge located at a 55-m range is shown in Figure S8.

References

1. Zyvoloski, G. A., Robinson, B. A., Dash, Z. V., & Trease, L. L. Summary of the models and methods for the FEHM application: A finite element heat and mass-transfer code. Rep. LA-13307-MS, Los Alamos Natl. Lab., Los Alamos, NM (1997).
2. Sander, R. *Henry's law constants. In: NIST Chemistry WebBook, NIST Standard Reference Database Number 69* [Linstrom, P. J., & Mallard, W.G. (eds.)] Available at: http://webbook.nist.gov (Accessed: 1 May 2013).
3. Sun, Y., & Carrigan, C. R. Modeling noble gas transport and detection for the Comprehensive Nuclear-Test-Ban Treaty. *Pure Appl. Geophys.* **171,** 735-750 (2014).
4. Millington, R. J., & Quirk, J. P. Permeability of porous solids. *Trans. Faraday Soc.* **57,** 1200–1207 (1961).
5. Carrigan, C. R., Heinle, R. A., Hudson, G. B., Nitao, J. J., & Zucca, J. J. Barometric gas transport along faults and its application to nuclear test-ban monitoring. Rep. UCRL-JC-127585, Lawrence Livermore Natl. Lab., Livermore, CA (1997).
6. Berkowitz, B. Characterizing flow and transport in fractured geological media: A review. *Adv. Water Resour.* **25,** 861-884 (2002).

7. Zubelewicz, A. *et al.* A mechanisms-based model for dynamic behavior and fracture of geomaterials. *International Journal of Rock Mechanics and Mining Sciences* **72,** 277-282 (2014).
